# Laser-Assisted Lingual Frenectomy: A Case Report

**DOI:** 10.7759/cureus.59412

**Published:** 2024-04-30

**Authors:** Prasad V Dhadse, Ruchita T Patil, Shrishti S Salian, Ranu R Oza, Sanehi D Punse

**Affiliations:** 1 Department of Periodontics and Implantology, Sharad Pawar Dental College and Hospital, Datta Meghe Institute of Medical Sciences, Wardha, IND

**Keywords:** haemostasis, less pain, tongue-tie, frenectomy, laser treatment

## Abstract

A lingual frenectomy is a surgical procedure aimed at addressing "tongue-tie" or ankyloglossia, where a strip of tissue restricting tongue movement is removed. Typically, this strip extends from the bottom of the mouth to the underside of the tongue. The procedure, often performed using a diode laser, offers several advantages including simplicity and safety for patients. It can significantly improve speech articulation and eating for individuals with ankyloglossia. This case report highlights the successful treatment of a female patient experiencing speech difficulties with diode laser therapy for tongue-tie.

## Introduction

A frenum is made up of folds of connective tissue, mucous membrane, and occasionally muscle fibers. The lingual frenum of the mandible, the buccal frenum in the premolar region, and the labial frenum in the midline of the upper and lower jaw are the most significant of these [1]. Ankyloglossia, often known as tongue-tie, is a common congenital condition of the sublingual frenulum that is characterized by restriction in the functions of the tongue. It is frequently linked to challenges with breastfeeding and can be treated surgically with a frenectomy which involves removing the frenulum and its attachment entirely [2]. In infants, the prevalence ranges from 4.4% to 4.8%, with a male-to-female ratio of 3:1. In clinical settings, the phrase has been used to characterize a variety of conditions, including a tongue that is fused to the floor of the mouth and a tongue that has limited mobility as a result of a thick, short lingual frenulum [3].

Untreated tongue-tie can contribute to weight gain problems in infants and failure to thrive in infancy. Apart from feeding difficulties, an untreated tongue-tie can lead to issues with dental alignment and speech defects [4]. Delay or decline in speech development is also linked to a short and fibrotic lingual frenum. Toddlers with tongue-tie frequently exhibit the pronunciation of consonants such as "t," "d," "n," and "l" in the development of frontal and lateral lisps. Lingual frenectomy typically involves a surgical incision made with a scalpel, electrocautery, or soft tissue lasers. The lingual tissue's morphology and anatomical placement render it susceptible to a variety of intraoperative and postoperative problems, despite the straightforward nature of the lingual frenectomy procedure [5]. This case report reveals the treatment of tongue-tie using a soft tissue diode laser.

## Case presentation

A 38-year-old female presented to the department of periodontics with difficulty in pronouncing a few words. The patient had no significant medical history and wasn't on any immunosuppressive, anti-diabetic, or anti-hypertensive drugs. The patient provided no relevant dental history. No gross asymmetry was observed during the extraoral examination, and there was bilaterally smooth and synchronized temporomandibular joint movement. The submental and submandibular lymph nodes on both sides were inspected, which were neither tender nor palpable.

**Figure 1:**
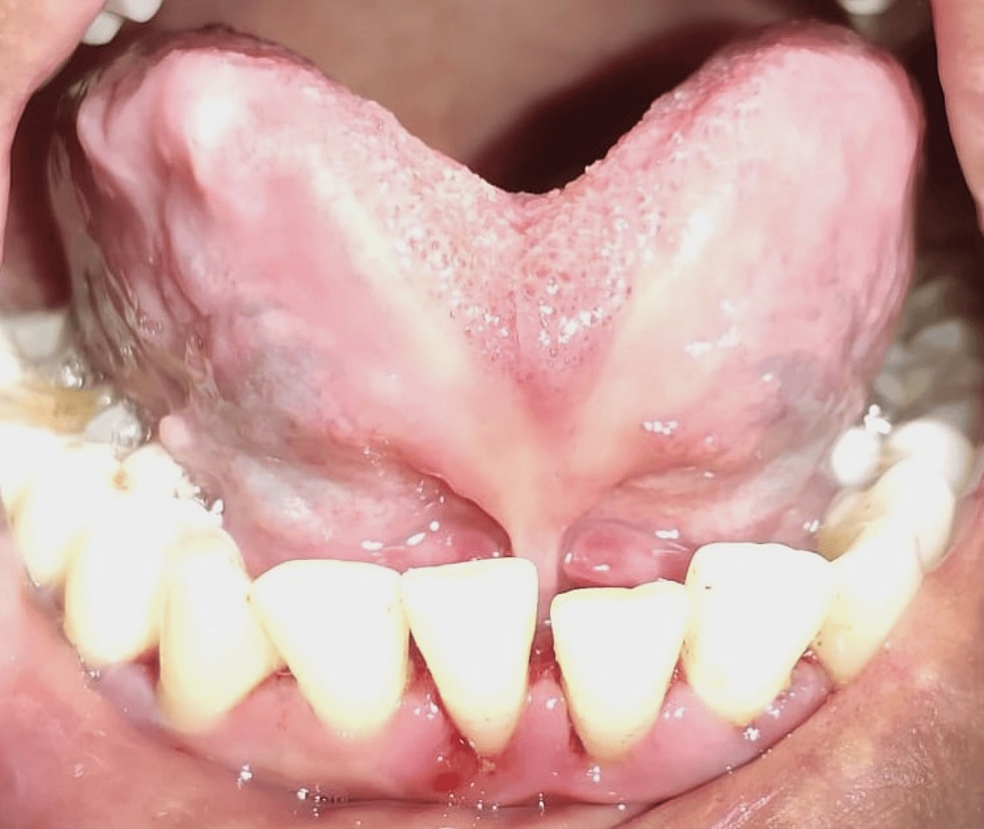
Preoperative photograph showing high lingual frenum attachment

**Figure 2:**
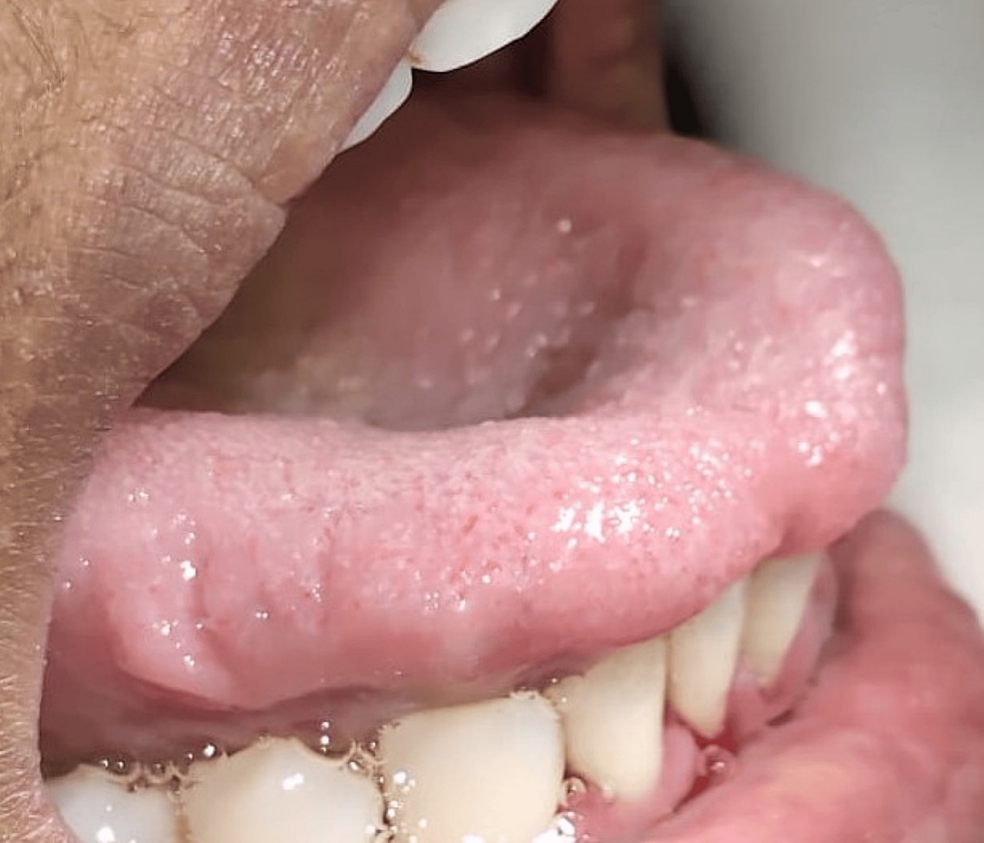
Preoperative tongue projection; the patient was unable to protrude the tongue

In intra-oral examination, it was noted that the patient had a high lingual frenal attachment (Figure 1). The patient was unable to place the tongue on the cingulum of the maxillary incisor and she was unable to project the tongue tip outside the mouth (Figure 2). Other intraoral findings include a root piece with 16 and spacing with lower incisors. 

After initial oral prophylaxis (ultrasonic scaling), the patient was then referred to the department of oral pathology for blood investigations, including hemoglobin (Hb), bleeding time (BT), and clotting time (CT) (Table 1).

**Table 1:** Hematological reports Hb: Hemoglobin; BT: bleeding time; CT: clotting time

Hematology	Patient values	Normal reference vales (for female)	Normal reference values (for male)
Hb%	12.0 gm%	11-14.5 gm%	12-15.5 gm%
BT	1 min 21 sec	1-3 min	1-3 min
CT	2 min 30 sec	1-5 min	1-5 min

**Figure 3:**
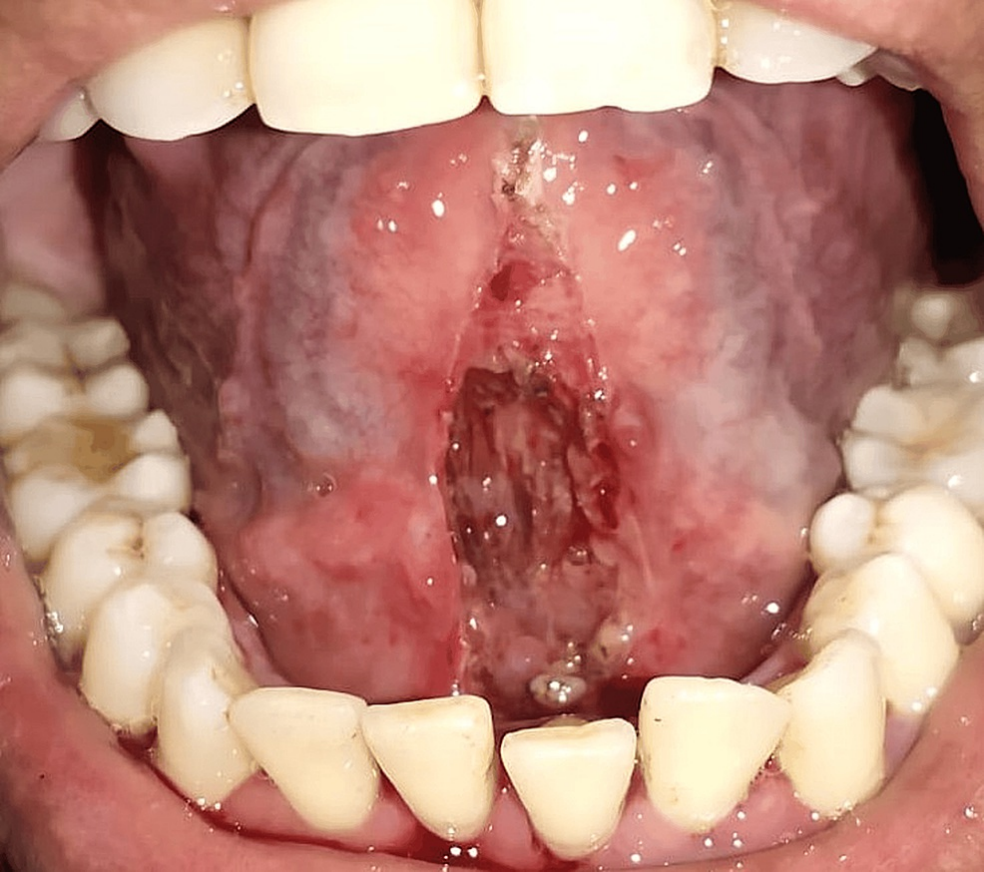
Frenectomy using laser

The patient was then recalled after eight days for a lingual frenectomy under all aseptic conditions and precautions. Under local anesthesia, the lingual frenum was held using a hemostat. A laser (EPIC X Diode Laser; Biolase, California, United States) tip was then applied to the bottom of the lingual frenum vertically and the fiberotomy was done (Figure 3).

**Figure 4:**
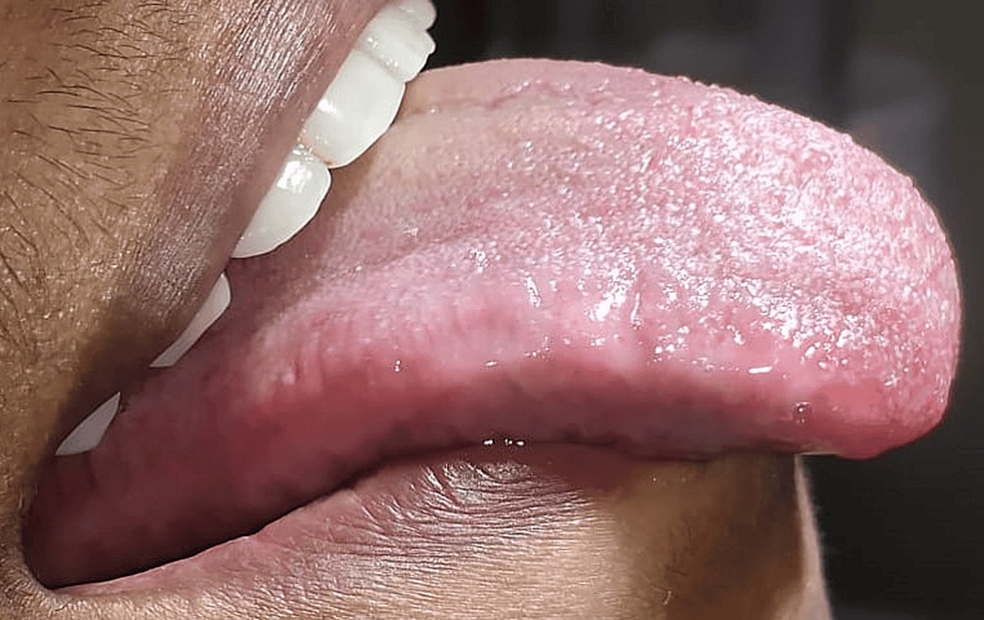
Postoperative projection of tongue after seven days

**Figure 5:**
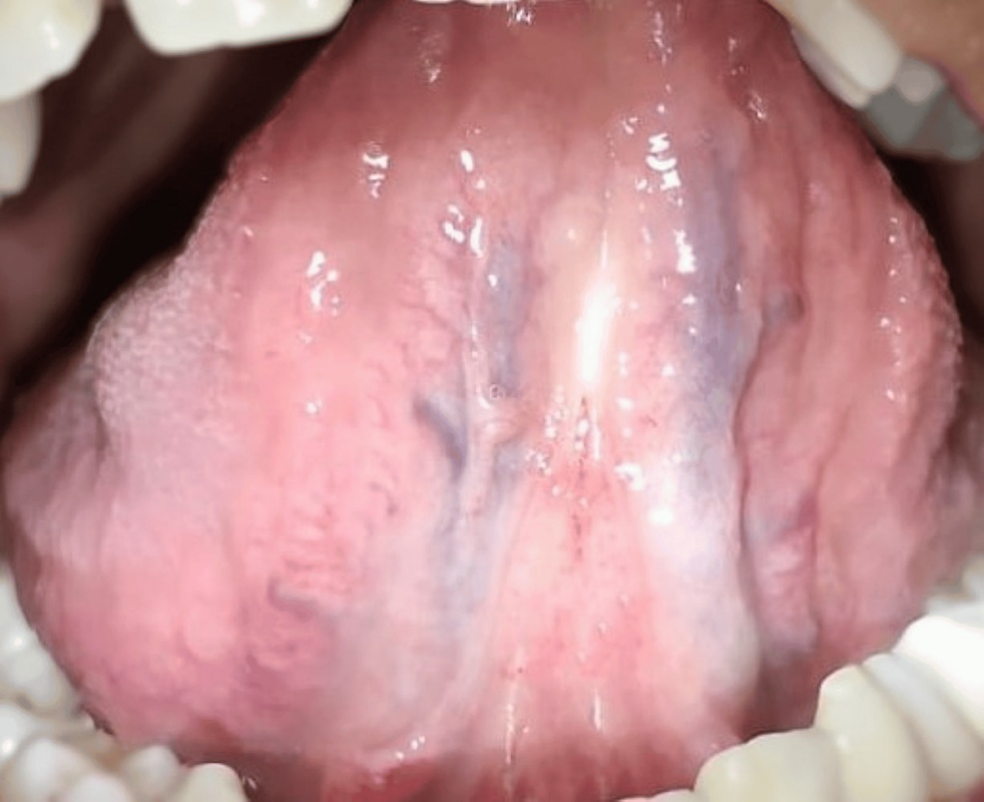
Postoperative healing after one month (base of the tongue)

After relieving fibrous attachment from the lingual frenum, hemostasis was achieved. Following the surgery, the patient was provided with postoperative instructions. She was given a prescription for postoperative pain medication. A follow-up appointment was scheduled for re-evaluation after a week, during which adequate tongue movement was observed upon examination (Figure 4). Complete healing was seen after one month postoperatively (Figure 5).

## Discussion

The abnormal insertion of the lingual frenulum, which compresses the mucosa, dense connective tissue, and occasionally the superior fibers of the genioglossus muscle, is the hallmark of ankyloglossia. Based on the distance of the insertion of the lingual frenum to the tip of the tongue, Kotlow’s classification of ankyloglossia is as follows: Class I, mild ankyloglossia 12-16 mm; Class II, moderate ankyloglossia 8-11 mm; Class III, severe ankyloglossia 3-7 mm; and Class IV, complete ankyloglossia <3 mm. In a tongue with normal function and range of movement, the interincisal distance by maximal mouth opening, while maintaining contact of the tongue tip to the posterior surface of the upper central incisor teeth should be >30 mm [6].

A normal range of tongue motion typically includes the ability for the tongue tip to extend beyond the mouth. Swiping the upper and lower lips with the tip of the tongue should be effortless and strain-free. The tongue should not be blanched when retruded anteriorly. A diastema between the mandibular central incisors should not be produced by the lingual frenum [7].

A gold standard treatment for tongue tie is frenectomy using a diode laser. Laser-assisted frenectomy in dental practices has grown in acceptance and popularity. Suturing is not needed in this procedure. Physiotherapy and phonetic instruction will begin immediately to aid the patient's recovery of tongue motion and phonation. There are several benefits of lingual frenectomy using a diode laser [8]. The primary developments documented in the literature include a decrease in postoperative pain and inflammation, bloodless surgery that results in the patient providing good psychological feedback, shorter surgical times, and technical simplicity [9].

In this present study, the use of a 980 nm diode laser allowed increased surgical precision and accuracy, thereby reducing unnecessary damage to underlying tissues, and the procedure was with no bleeding, resulting in improved visualization of the surgical field, eliminating the need for postoperative sutures, and shortening the operation time. It is probably the efficiency of the laser, which allows the sealing of lymphatic and blood vessels, that renders a bloodless surgical field [10].

In general, it is desirable to have no bleeding but, at the same time, it is also best to have the coagulated area as thin as possible. Localized denaturation of peri-vascular tissue, hemoglobin, and plasma proteins is the cause of hemostasis. Additionally, hemostasis is produced via coagulation through vessel wall contraction. With a low inflammatory response surrounding the tissues under surgical care, this hemostasis may explain the minimal fluid extravasation observed. The coagulated layer that forms over the raw area prevents visible bleeding but may take time to heal [11].

## Conclusions

The tongue is a highly vascular and dynamic structure. Laser-assisted frenectomy is the least complicated procedure option available for the treatment of tongue-tie. It also shows the best potential for minimally invasive dentistry outcomes. This report offers clinical evidence supporting the diode laser's superiority. By keeping the surgical field clean, it makes the procedure easier to perform and has minimal to no postoperative discomfort. For both the patient and the surgeon, this treatment offers a quick, safe, and effective way to treat ankyloglossia.
